# Draft genome sequence of multidrug-resistant *Enterobacter bugandensis* CUK-31 from wastewater of Himachal Pradesh, India

**DOI:** 10.1128/mra.00175-26

**Published:** 2026-05-29

**Authors:** Achhada Ujalkaur Avatsingh, Shilpa Sharma, Shilippreet Kour, Prem Prashant Chaudhary, Nasib Singh

**Affiliations:** 1 Department of Microbiology, Akal College of Basic Sciences, Eternal University 243975, Baru Sahib, Himachal Pradesh, India; 2 Laboratory of Clinical Immunology and Microbiology, Epithelial Therapeutics Unit, National Institute of Allergy and Infectious Disease, National Institutes of Health 573242 , Bethesda, Maryland, USA; Loyola University Chicago, Chicago, Illinois, USA

**Keywords:** *Enterobacter bugandensis*, wastewater, multidrug-resistant, India

## Abstract

We report the draft genome sequence of multidrug-resistant *Enterobacter bugandensis* CUK-31 isolated from wastewater in Shimla, Himachal Pradesh, India. Its genome harbors class C cephalosporinases, namely, *bla*_ACT-6_, *bla*_ACT-7_, *bla*_ACT-17_, and *bla*_ACT-49_, along with additional antibiotic resistance genes, and virulence genes.

## ANNOUNCEMENT

*Enterobacter bugandensis* is a clinically significant Gram-negative bacterium associated with sepsis and opportunistic infections (1, 2). Multidrug-resistant strains of *E. bugandensis* have been reported from foods (1), clinical samples (2), and aquatic ecosystems (3), indicating their significance in public health. Here, we report the draft genome sequence of *E. bugandensis* strain CUK-31 for determining its antibiotic resistance genes and virulence genes.

*E. bugandensis* CUK-31 was isolated from wastewater sample on 27 January 2024 from Shimla (30.986298° N, 77.620257° E), Himachal Pradesh, India. Sample was plated directly on eosin methylene blue (EMB) agar plates, followed by incubation at 35–37°C for 24 h. Streak plating was performed to get pure cultures on EMB agar plates at 35–37°C. The isolate was evaluated for phenotypic antibiotic susceptibility against 30 antibiotics belonging to 10 different classes by Kirby-Bauer disc diffusion method in accordance with Clinical Laboratory Standards Institute (CLSI) standard guidelines (4, 5). Results were interpreted as resistant, intermediate, and susceptible according to the breakpoints mentioned in CLSI document M100 (2021).

*E. bugandensis* CUK-31 was cultured in nutrient broth overnight at 37°C, and the genomic DNA was extracted by Xploregen DNA kit (Xploregen, Bengaluru, India) and quantified using Qubit 3 Fluorometer (Thermo Fisher Scientific, USA). Genomic library was prepared by enzymatic fragmentation of 100 ng of genomic DNA to a target size of 200–300 bp. The resulting fragments underwent end repair, 3′-adenylation, loop adapter ligation, and subsequent cleavage with uracil-specific excision reagent enzyme. Samples were purified using AMPure XP bead (Beckman Coulter, USA). DNA fragments were enriched by six-cycle PCR using NEBNext Ultra II Q5 Master Mix and Illumina primers. The final genomic library was quantified using Qubit 3 Fluorometer (Thermo Fisher Scientific, USA), and the fragment size analysis was performed by Agilent 2100 Bioanalyzer (Agilent Technologies, USA). Libraries were sequenced on an Illumina MiSeq sequencing system (150-bp paired-end). The quality of genome assembly sequences was evaluated by FastQC v0.12.1 (6), and the draft genome was assembled with Unicycler v0.5.1 (7). A total of 699 contigs were generated, which were subsequently ordered and oriented using scaffolding tool Multi-CSAR v1.0 (8) with the reference genome accession number GCF_035847925.1, resulting in 2 contigs in the final assembly. The assembled genome was annotated via the National Center for Biotechnology Information (NCBI) Prokaryotic Genome Annotation Pipeline v6.10 (9). Antibiotic resistance genes were predicted using CARD database v4.0.1 with perfect and strict match threshold (10) and ResFinder v4.7.2 with setting threshold for identification at 80% and minimum length at 60% (11). The virulence genes were predicted using the Virulence Factor Database (12) and MobileElementFinder v1.0.3 (13). All the analyses were performed using default parameters unless specified otherwise. The results are summarized in Table 1.

**TABLE 1:** Genomic characteristics of *Enterobacter bugandensis* strain CUK-31

Characteristic	Value	
Draft genome size (bp)	4,786,698	
N50/L50	4,737,968 (bp) / 1	
Number of raw reads	9,179,848	
GC content (%)	58.5	
Number of contigs	2	
Genome coverage	332.36×	
Total genes	4,679	
Total coding sequences	4,597	
Genes (protein coding)	4,527	
Number of rRNA genes	2	
Number of tRNA genes	74	
Number of ncRNA genes	6	
Pseudogenes	70	
CRISPR arrays	2	
Genome accession no.	JBTJCI000000000	
BioSample accession No.	SAMN48858669	
BioProject accession no.	PRJNA1271251	
SRA accession no.	SRR33806876	
Phenotypic antibiotic susceptibility	Resistant: ampicillin, amoxicillin/clavulanic acid, piperacillin, cefoxitin, ceftazidime, cefazolin, cefpodoxime, ceftriaxone, and imipenem Intermediate: cefotaxime and cefuroxime Susceptible: meropenem, cefepime, cefotetan, cefprozil, aztreonam, chloramphenicol, ciprofloxacin, norfloxacin, levofloxacin, tobramycin, gentamicin, amikacin, kanamycin, streptomycin, tetracycline, trimethoprim/sulfamethoxazole, azithromycin, piperacillin/tazobactam, and ampicillin/sulbactam	
Antibiotic resistance genes	β-lactams	*bla*_ACT-6_, *bla*_ACT-7_, *bla*_ACT-17_, and *bla*_ACT-49_
	Other antibiotic classes	*marA*, *adeF*, *oqxA*, *oqxB*, *emrR*, *emrB*, *rsmA*, *arnT*, *leuO*, *vanG*, *msbA*, *baeS*, *baeR*, and *fosA*
Virulence genes	*ompA, csgG, iroN,* and *nlpI*	

The genome of *E. bugandensis* strain CUK-31 harbored the antibiotic resistance genes encoding resistance to β-lactams and other antibiotic classes, as well as virulence genes. The genomic data will facilitate further insights into the resistance mechanisms and pathogenesis of this emerging pathogenic species and prove a valuable resource for comparative genomic studies.

## Data Availability

The whole-genome sequencing project has been deposited at NCBI under BioProject accession number PRJNA1271251, BioSample accession number SAMN48858669, and raw sequences have been deposited in Sequencing Read Archive under accession number SRR33806876. The assembled genome sequence is available at NCBI under accession number JBTJCI000000000.
